# Therapeutic Effect of Green Synthesized Silver Nanoparticles Using *Erodium glaucophyllum* Extract against Oral Candidiasis: In Vitro and In Vivo Study

**DOI:** 10.3390/molecules27134221

**Published:** 2022-06-30

**Authors:** Basem M. Abdallah, Enas M. Ali

**Affiliations:** 1 Al Bilad Bank Scholarly Chair for Food Security in Saudi Arabia, The Deanship of Scientific Research, The Vice Presidency for Graduate Studies and Scientific Research, King Faisal University, Al-Ahsa 31982, Saudi Arabia; eabdelkader@kfu.edu.sa; 2 Department of Biological Sciences, College of Science, King Faisal University, Al-Ahsa 31982, Saudi Arabia; 3 Department of Botany and Microbiology, Faculty of Science, Cairo University, Cairo 12613, Egypt

**Keywords:** oral candidiasis, silver nanoparticles, *Erodium glaucophyllum*, *C. albicans*, plant extract

## Abstract

Oral candidiasis (OC) is a fungal infection caused by an opportunistic fungi *Candida albicans*, which is found in the normal flora of healthy people. In this study, we examined the anti-*candidal* effect of green synthesized silver nanoparticles using leaf extract of *Erodium glaucophyllum* (EG-AgNPs) against *C. albicans* in vitro and in vivo. EG-AgNPs were synthesized for the first time using *E. glaucophyllum* extract and characterized by imaging (transmission electron microscopy (TEM), UV-VIS spectroscopy, zeta potential, X-ray diffraction (XRD), Energy dispersive x-ray analysis (EDX), and Fourier transform infrared spectroscopy (FTIR). A mouse model of OC was used for in vivo study. The agar well diffusion method showed the anti-*candidal* activity of EG-AgNPs against *C. albicans* with MIC 50 µg/mL. EG-AgNPs inhibited the dimorphic transition of *C. albicans* and suppressed the formation of biofilm by 56.36% and 52%, respectively. Additionally, EG-AgNPs significantly inhibited the production of phospholipases and proteinases by 30% and 45%, respectively. EG-AgNPs cause cytoplasm disintegration and deterioration of cell wall as imaged by SEM and TEM. Interestingly, EG-AgNPs did not display any cytotoxicity on the human gingival fibroblast-1 HGF-1 cell line at MIC concentrations. Topical treatment of the tongue of the OC mouse model with EG-AgNPs showed significant reduction in candidal tissue invasion, less inflammatory changes, and no tissue modification, in association with marked low scare and hyphal counts as compared to control group. In conclusion, our data demonstrated the potent inhibitory action of EG-AgNPs on the growth and morphogenesis of *C. albicans* in vitro and in vivo. Thus, EG-AgNPs represent a novel plausible therapeutic approach for treatment of OC.

## 1. Introduction

OC is a fungal infection of the human oral mucosal epithelium, caused mainly by the pathogenic fungus, *C. albicans* [1]. *C. albicans* is an opportunist fungus in oral mucosa, that under specific conditions could invade tissues by either endocytosis or direct penetration, and stimulate damage of the epithelium tissue [2]. Immunocompromised patients, such as those infected with immunodeficiency virus (HIV) as well as patients treated with immunosuppressant agents under chemotherapy are highly susceptible to be infected with OC, which could be transmitted to the blood stream and cause the systemic infection, candidaemia [3,4].

Several virulences are attributed to the pathogenicity of the *Candida* species. These include evasion of host defenses, biofilm formation, adherence to surfaces, morphogenesis, and production of proteolytic enzymes (SAPs), such as proteases and phospholipases [5].

*Candida* strains in both local and systemic candidiasis showed resistance to the most common commercially available antifungal drugs, in addition to the toxicity of these antifungal drugs [6,7]. Thus, candidiasis required more effective treatment with less toxicity. In this context, many metal nanoparticles and other NPs have been used as an alternative therapy for candidiasis due to antifungal resistance [8]. For example, Chitosan–silver–copper nanocomposites showed comparable antifungal activity against *C. albicans* with comparable results to amphotericin B [9]. Curcumin-loaded polymeric nanoparticles reduced *C. albicans* hyphae as an antimicrobial photodynamic therapy in murine models of oral candidiasis [10]. In addition, zinc Ferrite nanoparticle hybrid nanostructures significantly inhibited the biofilm formation of *C. albicans* cells in vitro [11]. Recently, we and others demonstrated the efficient inhibition of *C. albicans* by green synthesized AgNPs using plant extracts of *Calotropis gigantean*, *Lotus lalambenesis,* or *Artemisia annua* [12,13,14]. Despite the promising data on using nanoparticles as an antifungal drug against *C. albicans*, very few studies reported the in vivo effect of nanoparticles in animal models of candidiasis.

AgNPs have diverted the attention of the scientific community because of their highly accepted applications in biomedical fields. The unique properties of AgNPs are useful for cancer therapeutics because they resulted in improvement of the chemotherapeutic potential with lower systemic toxicity [15]. AgNPs attracted special attention for this particular field, and were successfully identified as efficient anti-tumor drug-delivery systems [16], acting either as passive [17] or active [18] nanocarriers for anticancer drugs. Recent studies reported the potential use of AgNPs as vaccine and drug carriers for specific cell targeting [19]. Additionally, the recent improvements in AgNPs biocompatibility and stability through surface modification recommend nano-structured systems depend on silver as specific and selective candidates for drug-delivery applications [20].

*E. glaucophyllum* (L.) Aip. belongs to the family *Geraniaceae andits*, a common herb in deserts, the Nile valley, and the western Mediterranean region [21]. *E. glaucophyllum* was used in traditional medicine as an oxytocic and astringent [22]. *E. glaucophyllum* possesses high contents of vitamin C, polyphenols, and carotenoids, and showed high antioxidant [23] and anti-inflammatory activities [24]. In addition, limited studies reported the anti-bacterial and antifungal activities of *E. glaucophyllum* [25].

For the first time, we used the leaf extract of *E. glaucophyllum* to biosynthesize AgNPs (EG-AgNPs), and examined its antifungal effect against *C. albicans* in vitro. Furthermore, the efficiency of topical treatments of EG-AgNPs was examined in a novel in vivo mouse model of OC. The importance of this study is to confirm the antifungal action along with the safety of EG-AgNPs use in vivo to treat OC.

## 2. Results

### 2.1. Biosynthesis and Characterization of EG-AgNPs

**Figure 1:**
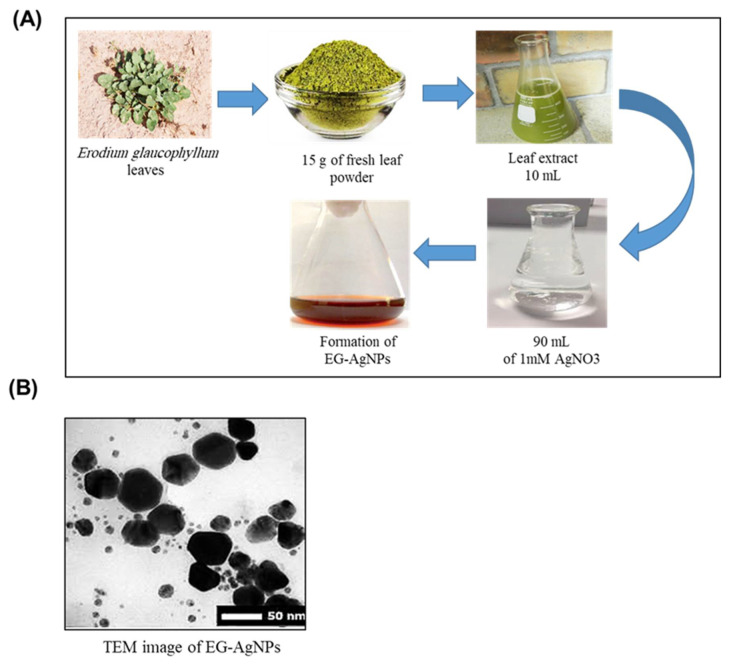
Biosynthesis of EG-AgNPs. (**A**) Green synthesis of EG-AgNPs. The color of solution changed from light yellow to dark brown as an indication of EG-AgNPs formation. (**B**) TEM micrograph of EG-AgNPs showing the size of AgNPs ranging from 50–100 nm of irregular spherical forms.

EG-AgNPs were biosynthesized using the leaf extract of *E. glaucophyllum*. The color change of the extract of *E. glaucophyllum* after the addition of silver nitrate from colorless to brown was identified (Figure 1A). This brown color designated the AgNPs production. The biosynthesis of EG-AgNPs was confirmed by TEM which displayed the morphology of the EG-AgNPs to be irregular spherical. Particles size and size distributions of the biosynthesized AgNPs using dynamic light scattering method are shown in Supplementary Figure S1. It is detected that the particles obtained have a good dispersion with diameters of approximately 50 nm. The size distribution of EG-AgNPs are described by intensity (Figure 1B).

**Figure 2:**
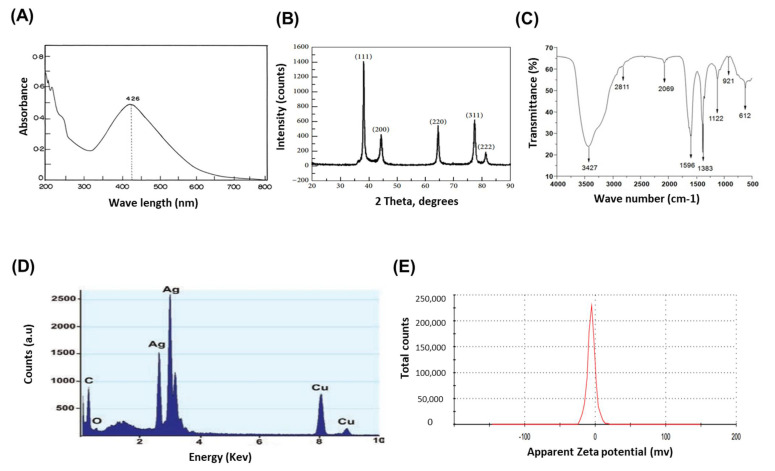
**Verification of EG-AgNPs formation.** (**A**) UV-Vis spectrum of green synthesized EG-AgNPs showing the absorbance peak at about 426 nm. (**B**) XRD spectrum of EG-AgNPs displayed distinct diffraction peaks indexed 20 (degree) values of (111), (200), (220), and (311), crystalline planes of cubic Ag. (**C**) FTIR spectrum of EG-AgNPs displayed peaks around at 3427, 2811, 1596, 1383, 1122, 921, and 612 cm^−1^. (**D**) EDX spectrum of EG-AgNPs. (**E**) Zeta potential of EG-AgNPs.

UV-visible spectrophotometry displayed a spectrum of surface plasmon resonance (SPR) at the 426 nm absorption peak (Figure 2A). Four intense peaks in the whole spectrum of 2𝜃 values ranging from 30 to 80 were revealed by the XRD pattern. The biosynthesized AgNPs were identified in the form of nanocrystals by XRD spectrum, with peaks at 2𝜃 values of 38.25°, 46.37°,64.60°, and 77.62°, corresponding to 111, 200, 220, and 311 planes for silver, respectively (Figure 2B). The possible interaction between functional groups and EG-AgNPs was studied by using the FTIR analysis. The spectral data were in the wavelength range of 4000–500 cm^−1^. Peaks around 3427, 2811, 1596, 1383, 1122, 921, and 612 cm^−1^ were reported by the FTIR spectrum. The peak at 3427 corresponds to –OH stretching of phenols and alcohols, the peak at 2811 accounts for C-H stretching of alkanes, the peak at 1596 corresponds to the bond stretch leads for N-H bend primary amines, the peak at 1383 corresponds to CH3CH bending of alkanes, the peak at 1122 assigned C-O bending, the peak at 921 accounts for C-Cl stretching of alkyl halides, and 612 corresponds to alkyl halides. (Figure 2C). EDX analysis showed a strong signal of the metallic silver region at 3 keV and confirms the formation of AgNPs. A weak signal for oxygen was observed which would correspond to the occurrence of Ag_2_O. Due to the carbon-coated copper grid for sample preparation, two moderate signals of copper and carbon were identified (Figure 2D). Lastly, the Zeta potential of −10 mV showed that the biosynthesized EG-AgNPs were stable, dispersed in the medium, and capped by negatively charged groups (Figure 2E).

### 2.2. Anti-Candidal Activity of EG-AgNPs

**Figure 3:**
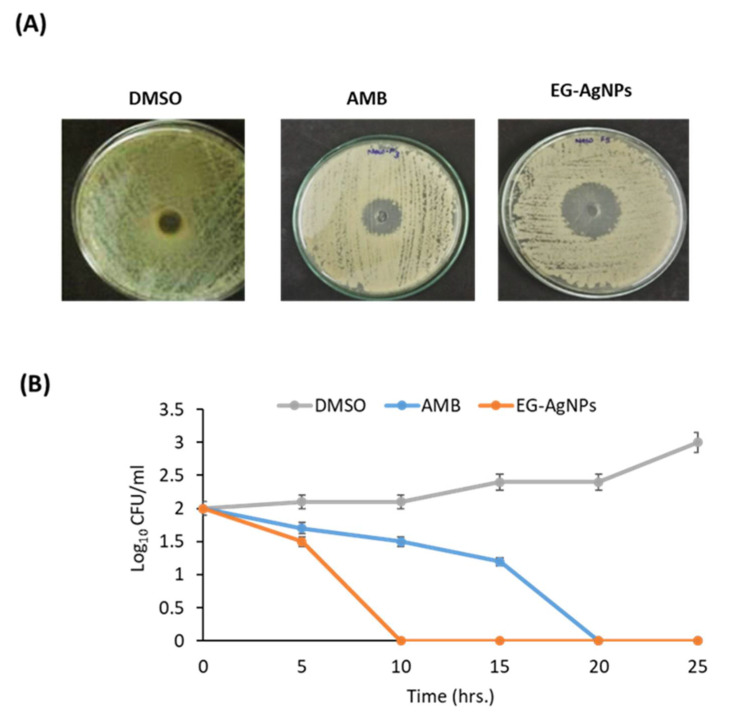
**Anti-candidal activity of EG-AgNPs.** (**A**) Agar well diffusion method showing the anti-candidal activity of EG-AgNPs (50 µg/mL). (**B**) Time kill curves of *C. albicans* inoculum (10³ CFU/mL) after exposure to EG-AgNPs (50 µg/mL) and amphotericine b (100 µg/mL). There was a reduction in the rate of cell growth when *Candida* cells were treated with EG-AgNPs compared to control cells.

We examined the anti-candidal effect of EG-AgNPs using the agar well diffusion method. The EG-AgNPs exhibited high anti-candidal activity compared to AMB where the diameter of inhibition zone (IZD) was 21 and 17 mm, respectively. (Table 1 and Figure 3A). The MIC values of EG-AgNPs and AMB were 50 and 100 µg/mL, respectively (Table 1). The time-kill assays showed the capacity of EG-AgNPs treatment to decrease fungal viability during the first five hours when applied at 100 µg/mL to *C. albicans* inoculum (10³ CFU/mL) (Figure 3B). At 24 h, the complete fungal load was eliminated, which was indicative of the higher efficiency of EG-AgNPs as a powerful anti-candidal agent (Figure 3B).

### 2.3. EG-AgNPs Inhibits the Virulence Factors of C. albicans

**Figure 4:**
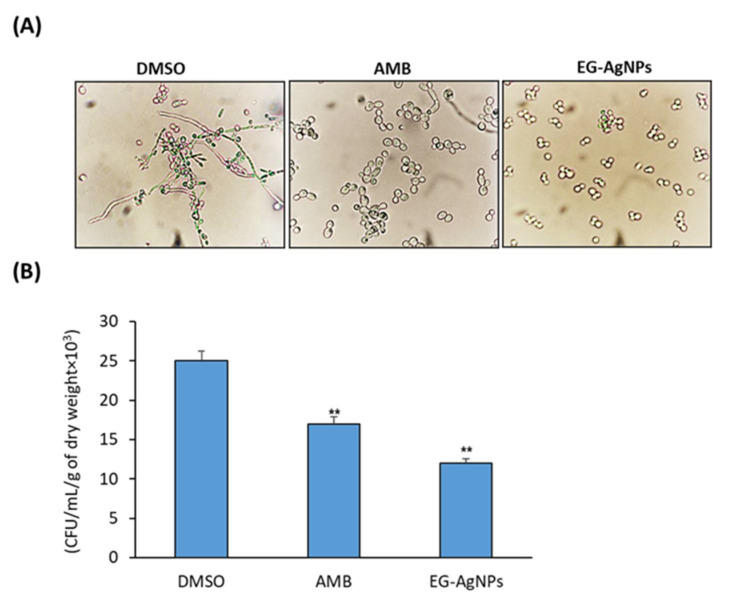
Effect of EG-AgNPs (50 µg/mL) on virulence factors of *C. albicans.* (**A**) Microscopic images of *C. albicans* dimorphic transition. Complete inhibition of dimorphic of *C. albicans* upon treatment with EG-AgNPs (50 µg/mL). (**B**) Treatment of *C. albicans* biofilms with EG-AgNPs (50 µg/mL). Viability of *C. albicans* biofilm expressed in CFU/mL/g of dry weight. (** *p* < 0.005, compared to control non-treated cells).

Untreated control (DMSO) of *C. albicans* showed higher hyphal growth after 6 h, whereas the capability of dimorphic transition of *C. albicans* was completely inhibited by 56.36% in the presence of EG-AgNPs at concentrations lower than their MIC values (25 µg/mL), as assessed by phase contrast microscopic examinations (Figure 4A and Table 2). In addition, biofilm assays revealed a significant reduction in fungal burden by 52% after treatment with EG-AgNPs at 50 µg/mL (MIC for *C. albicans*) as compared to untreated control (DMSO) (Figure 4B). The viability of the *C. albicans* biofilms treated with EG-AgNPs were lower than that of the positive control group, AMB (100 µg/mL) (Figure 4B).

### 2.4. EG-AgNPs Suppresses the Proteolytic Enzymes Activities

**Figure 5:**
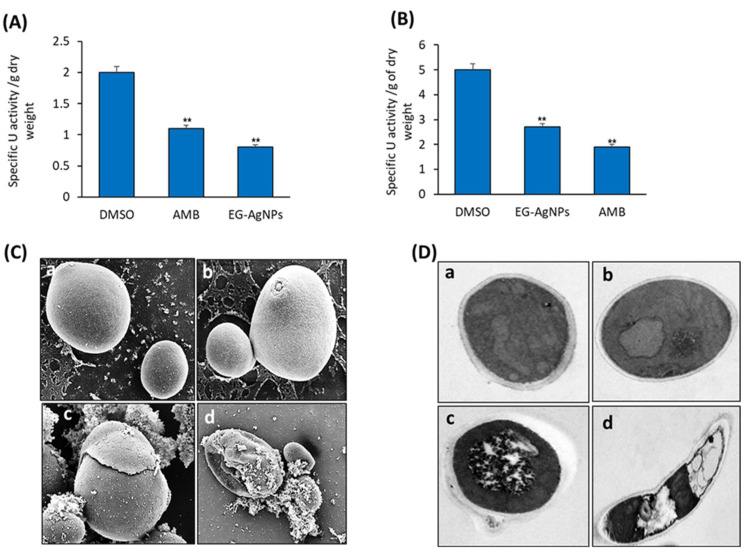
**Effect of EG-AgNPs on the proteolytic enzymes activities and morphology of *C. albicans.*** Inhibitory effect of EG-AgNPs (50 µg/mL) on the activity of proteinase enzyme (**A**), and phospholipase enzyme (**B**) activities of *C. albicans*. (**C**) SEM of *C. albicans* treated with (**a**,**b**) saline and (**c**,**d**) EG-AgNPs (50 μg/mL). Treated cells presented a rugged surface and detachment of an outer layer of the yeast cell wall. (**D**) TEM of *C. albicans* treated with saline (**a**,**b**) showing normal growth of *C. albicans* cells and (**c**,**d**) EG-AgNPs showing membrane retraction, swelling, and separation of an outer layer of the cell wall. Bar 250 = nm. (** *p* < 0.005, compared to control non-treated cells).

*C. albicans*-secreted enzymes were studied using the proteinase and phospholipase enzyme tests. Enzyme activities (specific activity units) were normalized by the total dry weight of cells (grams). EG-AgNPs (50 µg/mL) significantly reduced the enzyme activities of both proteinases and phospholipases in *C. albicans* by 30% and 45%, respectively (Figure 5A,B).

### 2.5. SEM and TEM Analysis of C. albicans Cells Treated with EG-AgNPs

Treatment of *C. albicans* with EG-AgNPs showed modifications in the morphology of fungal cells as compared to untreated controls. These morphological changes include cytoplasm disintegration, vacuolation, and perinuclear and granular nuclear alteration (Figure 5C(c,d)). The cell wall in many Candida cells was swollen and the outer layer appeared to be separated from the cell. TEM displayed masses of cellular debris and in some yeast cells, the cell wall appeared with a rugged surface (Figure 5C). A notable effect was the obvious separation of an outer layer from the yeast cell wall (Figure 5D(c,d)).

### 2.6. EG-AgNPs Exerts Therapeutic Effect toward OC in Mice

**Figure 6:**
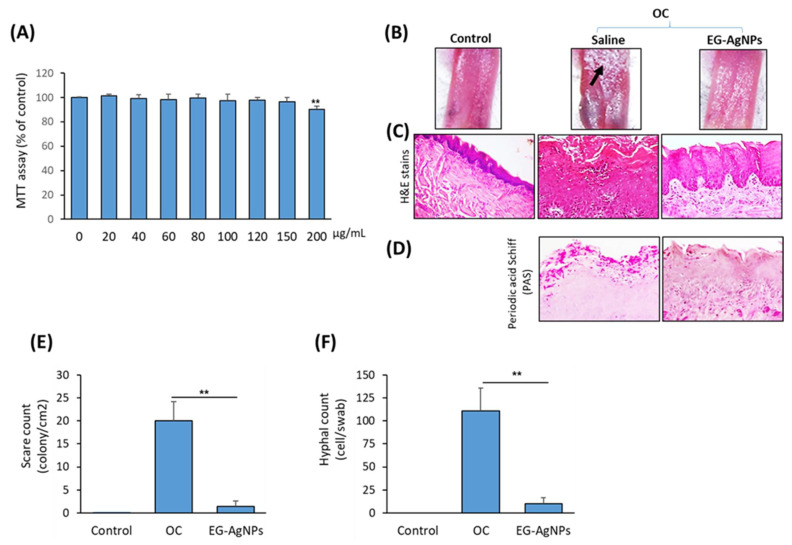
**Inhibitory effect of EG-AgNPs on OC in mice.** (**A**) Cytotoxicity of EG-AgNPs on human HGF-1 cell line. MTT assay was used to examine the effect of EG-AgNPs at different concentrations on the cell viability after 48 h of treatment. (**B**) photographic images of tongue dissected from control (non-infected) and OC mice treated with saline or EG-AgNPs (50 μg/mL). Histological analysis of tongue sections stained with H&E (**C**) and periodic acid shiff (PAS) (**D**). Tongue sections non-treated OC mice showed an irregular pattern of the covering epithelium. Several inflammatory changes were observed. EG-AgNPs-treated OC mice showed less inflammatory changes and hyphae invasion than control saline treated OC mice. Significant reduction in scares (**E**) and number of hyphal count (**F**) that recovered from oral cavity of OC mice treated with EG-AgNPs. (** *p* < 0.005, compared to control non-treated cells).

As shown in Figure 6A, EG-AgNPs showed no cytotoxicity on the human gingival fibroblast-1, HGF-1 cell line when used at concentrations up to 150 μg/mL. At concentrations of more than 150 μg/mL, EG-AgNPs started to exert significant cytotoxicity on HGF-1 cells (Figure 6A). Thus, EG-AgNPs can be used safely at their MIC value (50 μg/mL).

We further examined the therapeutic potential of EG-AgNPs in mouse models of OC. Mice infected with *C. albicans* in their tongues showed pathological symptoms of OC. These include patchy white areas of smooth mucosa and well-delineated atrophic areas on the dorsal of tongues as depicted in (Figure 6B). Interestingly, tongues treated topically with EG-AgNPs after *C. albicans* infection appeared normal and healthy without any symptoms of candidiasis compared to control (Figure 6B).

Histological studies using H&E and PAS staining demonstrated that *C. albicans* lesions could be observed near the oral epithelium of dorsal tongues of the non-treated group. Necrosis was also observed in the epidermal layer of stratified squamous epithelium, and the *Candida* appeared around necrotic tissue in the form of filament or yeast cells (Figure 6C,D).

In addition, the area of necrosis infiltrated with mononuclear cells was mainly macrophages and lymphocytes (Figure 6C,D). OC mice treated topically with EG-AgNPs displayed an intact tongue epithelium, with well-defined layers, and maintained papillary aspect. Furthermore, the connective tissue layer of treated tongue with EG-AgNPs had its integrity preserved, with regular fibroblasts and collagen fibers (Figure 6C,D). As shown in Figure 6E, treatment of OC mice with EG-AgNPs showed a significant reduction in the clinical scares of tongues. In addition, EG-AgNPs significantly reduced the number of hyphal counts that were recovered from the oral cavity as compared to the non-treated group (Figure 6F).

## 3. Discussion

*C. albicans* is a dimorphic fungus and it is a part of the normal microflora of oral cavity, where changes in the host ecological environment might help the dimorphic transformation of *C. albicans* into a pathogen that can invade the epithelial tissue and cause an infection [5]. Nowadays, the production of consistent, ecofriendly methods for the synthesis of nanoparticles is an important aspect of nanotechnology. In this study, we were the first to use *E. Glaucophyllum* in the biofabrication of AgNPs. We examined the inhibitory effect of EG-AgNPs on the growth, biofilm and morphological transformation of *C. albicans* in vitro and its therapeutic potential on OC in vivo.

In this study, the biosynthesized EG-AgNPs was confirmed by an intense peak at 426 nm due to surface plasmon resonance. This data is in agreement with other reported biosynthesized AgNPs [26,27]. The crystalline nature of the EG-AgNPs was approved using the XRD pattern analysis and the diffractogram was at the 2*θ* value of 38.25°. This due to the organic components of the *E. Glaucophyllum* leaf extract, which were responsible for the stabilization of the AgNPs and the bio-reduction of the Ag ions [28,29]. According to FTIR results, the strong absorption peaks at 3427 and 1596 cm^−1^ reveal O–H and C–C stretching vibrations of aromatic compounds, respectively. OH stretch might be attributed to the phytochemicals such as alkaloids, terpenoids, flavonoids, and phenolics which are abundantly presented in the leaf extract [30]. We suggested that the biosynthesized AgNPs were capped by biomolecules involved in AgNPs formation [31]. EDX analysis of the synthesized EG-AgNPs displayed a typical optical absorption peak at 3 keV, which confirmed the presence of elemental Ag in the form of AgNPs [32]. After the end of reaction, the product was recovered, obtaining 1.53 g of powder with >99% silver content as determined by atomic absorption spectroscopy. The zeta potential is important for understanding the stability of nanoparticles, as it is an indicator of surface charge potential. Zeta potential values of EG-AgNPs were −10 mV, and thus EG-AgNPs were considered very stable in the dispersion medium, based on the reported zeta potential for other nanoparticles, which are higher than +30 mV or lower than −30 mV are [33].

Several studies showed that the size, morphology, stability, and properties of the AgNPs are greatly affected by the experimental conditions, the kinetics of interaction of metal ions with reducing agents, and adsorption processes of stabilizing agents with metal nanoparticles [34]. The shape and size of produced AgNPs depend on experimental conditions such as: temperature, the concentration of silver precursor, pH of the solution, the molar ratio between PVP (the capping agent) and AgNO_3_ (silver precursor), the strength of chemical interaction between PVP and various crystallographic planes of silver [35], reducing agents, and the method used (chemical, physical, or biological). Furthermore, by controlling the chemical and redox environment of the initial seed particle, the shape of the produced nanoparticles can be changed. A change in seed concentration [36], surfactant [37], pH [38], temperature [39], and concentrations [40], can have severe effects on the shape and size of the produced nanoparticles.

Our results identified the MIC for EG-AgNPs to be 50 µg/mL with higher anti-candidal action. This effect could be explained due to the presence of carotenoids, polyphenols, flavonoids, and tannins contents in the extract of *E. glaucophyllum* [23,24]. In addition, the phenolic components of *Erodium* spp. include tannins, catechins, gallic, and ellagic acids that support their antimicrobial properties against pathogenic microorganisms [21,22]. In this context, the AgNPs biosynthesized using the extract of *Lycopersicon esculentum*, which showed antifungal action against *C. albicans*, *C. parapsilosis*, and *C. glabrata* with MIC of 8 µg/mL [41]; however, AgNPs biosynthesized using the extract of *Artemisia annua* displayed MIC against *C. albicans*, *C. tropicalis*, and *C. glabrata* and ranged between 80 and 120 μg/mL [14]. Similarly, the biosynthesized AgNPs using the seed extract of *Syzygium cumini* displayed anti-candidal activity at 0.125–0.250 mg/mL [42]. AgNPs biosynthesized by beech bark extract showed MIC of 0.03 mg/mL against *C. albicans* [43]. Furthermore, AgNPs synthesized by *Caesalpinia ferrea* extract displayed MIC values in the range of 156.25–1,250 µg/mL against *C. albicans*, *C. glabrata*, *C. kruzei*, and *C. guilliermondii* [44]. Additionally, we recently described the anti-candidal activity of AgNPs bio-fabricated by *Calotropis gigantea* leaf extract and *Lotus lalambensis* root extract against *C. albicans* with MIC 50 μg/mL [12,13].

Several studied described the anti-candidal potential of AgNPs obtained by physical and chemical synthesis methods. For example, AgNPs synthesized by microwave-assisted techniques demonstrated a potent dose-dependent inhibitory effect on biofilm formation of *C. albicans*, with an IC50 of 0.089 ppm [45]. Additionally, chemically synthesized AgNPs using sodium borohydride as the reductant displayed anti-candidal potential with MIC of 62.5 μg/mL [46]. Furthermore, silver colloidal nanoparticles synthesized by chemical reduction showed excellent antifungal potential against *C. albicans* with MIC of 20 μg/mL [47]. Chemically synthesized AgNPS using pullulan displayed antifungal potential against *C. albicans*, *C. glabrata*, and *C. parapsilosis* with 98% inhibition at 8 mM Ag [48]. It should be noted that due to a lack of standardization, the antimicrobial potential of AgNPs identified in publications is inconsistent. This might be due to the MIC values being highly dependent on the method of nanoparticles synthesis, their physico-chemical properties, methods for MIC evaluations, and the density of the target microbial inoculum [49].

Several mechanisms were designated to detect the mode of anti-candidal action of AgNPs. These comprise the capability of AgNPs to damage the membrane permeability barrier and to destruct the membrane lipid bilayers, resulting in the leakage of ions, followed by formation of pores, and disintegrating the electrical potential of the membrane. Furthermore, AgNPs were described to block the cell cycle at the G2/M phase in *C. albicans* [50], which lead to an increase in the production of reactive oxygen species (ROS), and reduced the activity of metal-based antioxidant enzymes [51].

Dimorphic transition is considered the most critical factor in prompting epithelial invasion, which triggers the degradation of epithelial cell junction proteins [52]. The formation of hyphae is a significant property of *C. albicans* that plays an important role in adhesion and biofilm formation, which is necessary for colonization and pathogenesis of *C. albicans* [53,54]. Therefore, the blocking of morphogenesis from yeast to a filamentous form could means controlling the infection. Our results displayed the inhibitory activity of EG-AgNPs on the morphogenesis of *C. albicans* between yeast and filamentous forms. The precise mechanism of dimorphism suppression by AgNPs is not clear. AgNPs could also affect Ras-mediated signal pathways in *C. albicans* by suppressing the expression of the hyphal inducer gene (TEC), cell elongation gene (ECE1), and yeast to hyphal transition genes (TUP1 and RFG1), which are necessary for morphogenesis. Additionally, the morphogenesis of *C. albicans* is disturbed by disrupting the physiological structure and phospholipid content of cell membrane [55].

The formation of biofilms also play a major role in pathogenicity [56]. Cells in a biofilm have higher resistance to antifungal agents and the host immune system, mainly due to less penetration of antifungal drugs [57]. Biofilms increase adherence to surfaces and are important for stable colonization in host tissues [58]. Our results displayed that EG-AgNPs inhibited biofilm formation by 52%. In this context, Lara HH et al., in 2015, demonstrated a dose-dependent and potent inhibitory effect of AgNPs on biofilm formation, which were calculated as IC_50_ of 0.089 ppm. AgNPs biosynthesized by *Anabaena variabilis* extract resulted in 62.5% biofilm inhibition and degradation at a concentration of 25 µg/mL [45,59].

The exact mechanism by which AgNPs inhibit biofilm formation is unknown [60], suggesting that anti-biofilm effect was owing to the AgNPs superficial binding and increases penetration into the biofilm, disturbing the lipidome of cell membranes. Another possible mechanism includes the suppression of hyphal formation by AgNPs and the rupture of the cell wall, which allows both yeast and filamentous *Candida* spp. to survive [45,60].

Proteolytic enzymes, such as proteinases and phospholipases, are enzymes associated with hyphal development, tissue degradation, and host invasion, which are key factors associated with the pathogenicity of *C. albicans* [52]. Proteinases secreted by *C. albicans* have a destructive effect on the epithelial tissue during mucosal infections, as these enzymes facilitate hyphal invasion via increase the degradation of E-cadherin, a major protein present in epithelial cell junction [61]. Our results showed that AgNPs had resulted in a significant reduction in enzyme activities of both proteinases and phospholipases by 45% and 30%, respectively. Similar results reported that biosynthesized AgNPs significantly inhibit the production of phospholipases activity by 82.2, 75.7, 78.7, 65.8, and 62.5% in *C. albicans*, *C. tropicalis*, *C. dubliniensis*, *C. krusei*, and *C. parapsilosis,* respectively [42]. Hamid S. et al. found that AgNPs (100 µg/mL) biosynthesized by *Aspergillus* spp inhibit the secretion of proteases in *C. albicans* [62]. It is also reported that *Candida* cells can recognize AgNPs as stress signals and thus activate the quorum sensing mechanism that resulted in down regulation of different proteinases mRNA expression. We proposes that one probable antifungal mechanism of action of AgNPs includes the inhibition of the protease secretions [63].

Our results confirmed that EG-AgNPs treatment of *C. albicans* cells caused significant alterations in the cell wall and membrane as observed by SEM and TEM. Similarly, major modifications in the ultrastructure of *C. albicans* cells were previously described for biosynthesized AgNPs [42,45]. These changes might be caused by the interference of AgNPs within the cell wall. AgNPs disrupt the cell wall and cell membrane of *C. albicans* and cause severe alterations, resulting in blebs on the surface and cell collapse. AgNPs could affect the membrane permeability barrier by disturbing the membrane lipid bilayers, resulting in the leakage of ions and other materials. This membrane leakage is accompanied by the formation of pores and dissipates the electrical potential of the cell membrane [50]. Radhakrishnan VS. et al. reported that AgNPs modified surface morphology, cellular ergosterol content, cellular ultrastructure, membrane fluidity, and fatty acid composition, which are important for morphogenesis [64].

As compared to chemically synthesized AgNPs, the green synthesized AgNPs using different plant extracts showed antimicrobial activity with low cytotoxicity in human and animal cells when applied at MIC values [12,13,65,66,67]. Shape, size, and concentration are important factors that affect the toxicity level of AgNPs. In support of this notion, small sized nanoparticles showed high toxicity against cancer cells [68], and small-sized AgNPs (10 nm) showed to induce apoptosis in MC3T3-E1 cells, as compared to large-sized AgNPs (50–100 nm) [69]. Thus, the size range of EG-AgNPs (50–100 nm) could be attributed to their low cytotoxicity on the HGF-1 cell line. In addition, the reported anti-inflammatory and antioxidant activities of *E. glaucophyllum* could be a benefit by reducing the cytotoxicity of EG-AgNPs as mentioned previously for green synthesized AgNPs using different *Lotus* species, for example [13,70,71].

Topical application of EG-AgNPs on the dorsum of the tongue of OC mice showed significant reduction in the viability of *C. albicans*. This results suggested the direct inhibitory action of EG-AgNPs on the growth of *C. albican* in vivo. Our recent in vitro studies support this direct effect, in which the green synthesis AgNPs using leaf extract of *Lotus lalambensis*, or *Calotropis gigantean* were shown to significantly inhibit the growth and the dimorphic transitions between yeast and filamentous forms of *C. albicans* [12,13]. Green- sensitized AgNPs using the leaf extract of *Polyalthia longifolia* were shown to inhibit the hyphae formation of *C. abicans* by downregulating the expression of genes involved in the transition of yeast-to-hyphal form [72]. In this context, several studies reported the inhibitory effect of green-synthesized AgNPs on the adhesion and biofilm formation of *C. albicans* [73,74,75]. Thus, it is plausible that, the therapeutic effect of EG-AgNPs on controlling the infection of candidiasis is mediated by blocking the transition between yeast and the hyphal form of *C. albicans.*

It is difficult to develop an ideal model of oral candidiasis. As it is also the case for other models of infectious diseases, no single model of oral candidiasis is able to answer all questions, and each has its own limitations. Therefore, significant variability from one experiment to another has to be minimized in the future by defining the minimum criteria to ensure reliable support. Additionally, one of the limitations of our study is the lack of comparison between using EG-AgNPs alone with the use of EG-AgNPs in a combination with plant extract or antifungal drug.

## 4. Materials and Methods

### 4.1. Plant Collection and Extraction

*E. glaucophyllum* was collected in matured form in April 2021 from the area of Shudqum (Al Hassa- El Dammam road), eastern providence, Saudi Arabia, and the plant was classified at the Cairo University herbarium with the voucher number T5F9. The plant was washed with distilled water and placed in cool and dry place to dry for ten days. The plant was ground into a fine powder. In total, 100 g of plant powder was shacked in 300 mL of 75% methanol. The methanol plant extract was dried in a rotary evaporator, and filtered through Whatmann NO. 1 filter paper. The plant extract was kept at 4 °C to be used in further experiments [76].

### 4.2. Preparation and Characterization of EG-AgNPs

#### 4.2.1. Green Synthesis of EG-AgNPs

A total of 220 mL of plant extract was added to 110 mL of 10 mM silver nitrate (AgNO3) solution. The solution was stirred for 24 h at room temperature. AgNPs were collected by centrifugation at 12,000 rpm for 15 min at 4 °C. The pellet was redispersed in water, centrifuged, and lyophilizated to obtain Eg-AgNPs powder.

#### 4.2.2. UV-Visible Spectrophotometry

One hundered μL of Eg-AgNPs was mixed with 1 mL of deionized water and examined in UV Visible spectroscopy using the Perkin Elmer UV-visible absorption spectrophotometer with a resolution of 1 nm between 200 and 800 nm. The scanning speed of 300 nm/minutes was used. The capacity of plant extract to reduce pure Ag ions to form AgNPs was measured by UV-Visible spectrum of the reaction medium.

#### 4.2.3. TEM

Samples were prepared by drop coating EG-AgNPs solutions onto carbon-coated copper TEM grids. After two minutes, the extra solution was removed and the grid was left to dry. The TEM were performed on JEOL (JEOL-JEM 1400, Freising, Germany).

#### 4.2.4. X-ray Diffraction Analysis (XRD)

XRD measurement of EG-AgNPs was performed using a Cu-K𝛼 radiation source in a wide range of Bragg angles (2𝜃) at a scanning rate of 0.388/min. The powder diffractometer (PANalyticalX per PRO model X-ray diffractometer) was used at a current of 30 mA and voltage of 50 kV.

#### 4.2.5. Fourier Transforms Infrared (FTIR) Spectroscopy

EG-AgNPs solution were centrifuged at 5000 rpm for 30 min. Pellets were resuspended in distilled water and lyophilized to create fine particles (KBr pellets). The measurements were carried out from 4000 to 500 cm^−1^ using a Perkin Elmer infrared spectroscopy.

#### 4.2.6. Energy Dispersive X-ray Spectroscopy (EDX)

EDX was carried out by Energy Dispersive X-ray analysis (EDX). A double-sided carbon-coated glass cover slip was used, where the lower side was used to fix the stab, whereas the upper side sample was loaded and examined by EDX (Model No. INCA 200, Oxford, UK).

#### 4.2.7. Zeta Potential

The zeta potential of AgNPs was calculated using the Zetasizer Nano Instrument (Malvern). The analysis of zeta potential taken at 25 °C with a 90° angle.

#### 4.2.8. Dynamic Light Scattering

The particle size distribution of AgNPs was assessed by dynamic light scattering (DLS) measurements directed with a Malvern Zetasizer Nanoseries compact scattering spectrometer (Malvern Instruments Ltd., Malvern, UK). Data obtained were analyzed using Zetasizer software.

### 4.3. Organism and Growth Conditions

*C. albicans* was formerly isolated from the oral cavity and identified by our group as described [13]. The number of blastospores/mL of suspension were evaluated by haemocytometer counting, and an appropriate volume of suspension was added to 100 mL of broth to produce an initial concentration of 10^6^/mL blastospores.

### 4.4. Anti-Candidal Activity of EG-AgNPs

Anti-candidal activities and a minimum inhibitory concentration (MIC) of EG-AgNPs were carried out by the agar well diffusion and broth dilution methods [77]. Before use, the solution of AgNPs was prepared by dissolving AgNPs in 5% dimethylsulfoxide (DMSO, 1000 µg/mL). The sample was sonicated at 30 °C for 15 min. A standard antifungal agent, amphotericine b, at 5 µg/well was used as a positive control, whereas 5% DMSO was used as a negative control. The culture of C. albicans was diluted to 1 × 10^6^ CFU and the anti-candidal activity of the AgNPs and AMB were evaluated by measuring the diameter of inhibition zones after 48 h of incubation at 28 °C. The minimum inhibitory concentration (MIC) of the AgNPs and AgNPs/CG were determined using the bi-fold serial dilution method [78]. Fifty microliters of different concentrations of AgNPs and AMB (6.25–400 µg/mL) were used for the MIC assay. The MIC was identified as the lowest concentration of the EG-AgNPs that did not show any growth of fungal colonies on the agar plates. MIC values were expressed as µg/mL.

### 4.5. Time-Kill Assay

*C. albicans* inoculum (10³ CFU/mL) was prepared in RPMI 1640 medium using spectrophotometric methods according to NCCLS protocol [79]. EG-AgNPs at their MIC concentration was added to the inoculum concentrations in 96-well plates. Other tested compounds included AMB (100 μg/mL) (positive control) and 5% DMSO (negative control). Test solutions were placed in a shaker and incubated at 35 °C, for defined time intervals (0, 1, 2, 4, and 24 h) and 100 μL aliquots were removed. For colony counting, tenfold serial dilutions were performed on all samples, and 10 μL aliquot from each dilution was plated on a SDA plate. The number of CFU on each plate was determined after incubation at 35 °C for 48 h.

### 4.6. C. albicans Hyphal Formation in Liquid Media

*C. albicans* grown overnight were inoculated in RPMI-1640 medium supplemented with either AMB, EG-AgNPs or DMSO at 37 °C for 24 h with shaking. Aliquots of *Candida* cells were harvested after 24 h and examined using the bright field with Digital Cell Imaging System (Logos Bio Systems, Anyang, Korea) [80].

### 4.7. Biofilm Assay

An inoculum of 1x10^6^ CFU/mL of *C. albicans* was prepared in 24-well plate using Yeast Nitrogen Base Medium (Difco) (Sigma-Aldrich, Burlington, MA, USA) supplemented with 50 mM of glucose and incubated at 37 °C in 5% CO_2_ for 24 h to start biofilm growth, as described by [81]. In total, 1 mL of inoculum was added to each well. After 24 h of incubation, the biofilms were treated with EG-AgNPS at their MIC concentration. Biofilms were washed daily with Phosphate Buffer Solution (PBS) and refilled with fresh medium. Adhered biofilms were collected after 72 h, suspended in PBS, and centrifuged at 10,000 rpm for 5 min. The biomass (dry weight) of each biofilm sample was placed in a speed vacuum to dry for 40 min. For CFU determination, each sample of biofilm was suspended in 1 mL of PBS and 20 μL of the suspension plated on SDA plates, at 37 °C in 5% CO_2_., The number of *C. albicans* colonies was counted after 24 h of incubation.

### 4.8. Proteinase and Phospholipase Enzyme Activities

Proteinase and phospholipase enzyme activities were determined as described by [82]. *C. albicans* cells were treated with EG-AgNPs as mentioned previously. The proteinase enzyme activity was measured by mixing the supernatant with 1% azocasein at 1:9 (*v*/*v*) for 1 h at 37 °C. The reaction stopped by adding 500 μL of 10% trichloroacetic. After centrifugation, 500 μL of the supernatant was added to 500 μL of NaOH, and incubated for 15 min at 37 °C. Absorbance was determined at an absorbance of 440 nm [83]. The phospholipase enzyme activity was analyzed by mixing the supernatant with phosphatidylcholine substrate for 1 h at 37 °C. The values of the enzyme were measured at an absorbance of 630 nm [84].

### 4.9. Morphological Modifications of C. albicans

#### 4.9.1. SEM

Yeast cells subjected to EG-AgNPs were fixed in the glutaraldehyde-cacodylate buffer solution for 1 h at RT, washed with cacodylate buffer 0.1 M, and the pellet post-fixed in the same buffer with 1% osmium tetroxide for 1 h. The cells were washed and treated with 1% tannic acid in water for 30 min. Pellets were incubated with 1% osmium tetroxide for 30 min and washed with water. Pellets were immersed in ethanol in a 50% to 100% gradient for dehydration. The samples were dried with CO_2_ and placed on copper grids to be imaged by SEM in a Hitachi S-5500 (Hitachi High-Technologies Europe GmbH, Krefeld, Germany) [45].

#### 4.9.2. TEM

*C. albicans* were incubated in with EG-AgNPs (50 μg/mL). Cells were fixed with 2.5% glutaraldehyde in sodium cacodylate buffer, and post-fixed in the same buffer with 1% osmium tetroxide for 2 h. The pellets were embedded in Spurr resin after dehydration by ethanol gradient (70–100%). Then, 0.25% toluidine blue was used to stain the semi-thin cuts (300 nm) for observation in an optical microscope (Axiophot Zeiss, with plan achromat objective), followed by ultra-thin cuts (70 nm) using a Porter Blum MT-2 ultra-microtome (Sorval, Liverpool, NY, USA). Samples were imaged in JEOL 100CX TEM (JEOL USA Inc., Peabody, MA, USA).

### 4.10. Cell Culture and Cytotoxicity Assay

The human gingival fibroblast-1 HGF-1 cell line was obtained from ATCC (#CRL-2014). Cells were cultured in high-glucose Dulbecco’s modified Eagle’s medium (DMEM; Sigma-Aldrich, MO, USA) supplemented with 1% penicillin/ streptomycin, 10% heat-inactivated fetal bovine serum (FBS) (Sigma-Aldrich), and 4 mM L-glutamine (Sigma-Aldrich) (Gibco Invitrogen, Carlsbad, CA, USA). Cells were cultured at 37 °C in a 5% CO_2_ atmosphere with the medium changed every 2 days.

The effect of EG-AgNPs on cell viability of the HGF-1 cell line was performed using the MTT cell proliferation assay kit (Sigma-Aldrich) according to the kit instructions. Cells were treated with EG-AgNPs at different concentrations (ranged from 0–200 µg/mL) in 96-well plates for 48 h. A medium containing 0.5 mg/mL MTT was used to replace the cultured medium to metabolize to formazan. An wELISA plate reader was used to measure the optical density at 550 nm using an [13].

### 4.11. Experimental Animal Design

We used 36 female Swiss albino mice (7–8 weeks of age) obtained from National Cancer Institute Cairo University, Egypt. The Cairo University, Faculty of Science, Institutional Animal Care and Use Committee (IACUC) (Egypt), and (CUFS/F/10/13) approved the procedure of in vivo OC experiments. Mice were housed at five animals per cage and maintained in a controlled temperature of 25 ± 2 °C and 12 h dark/light cycle as standard. Food (Altromin Spezialfutter GmbH & Co. KG, Lage, Germany) and water were provided ad libitum.

*C. albicans* cells were grown for 24 h in 50 mL of modified glucose salts biotin medium at 30 °C with aeration. Cells suspended in saline to the required concentration.

The protocol of the OC model in mice was carried out according to the method of [10,85]. Briefly, mice were immunosuppressed by injecting prednisolone subcutaneously at the dose of 100 mg/kg on days 1, 5, and 10. Tetracycline hydrochloride (Sigma) at a dose of 0.08 % was provided to the animals in the drinking water during the experimental period.

To induce candidiasis, animals were anesthetized by intramuscular injection of chlorpromazine chloride (100 uL of 2 mg/mL). Then, sedated animals were orally infected after 2 days of immunosuppression with 5 × 10^6^ cells mL^−1^ of *C. albicans* (in modified glucose salts medium) using a cotton swab rolled on the dorsum of the tongue. The infection was verified using visual examination.

For treatment: mice were divided into three groups (n = 12 mice/group) as follows: group 1: control group (non-infected); group 2: infected mice with *C. albicans* and treated with saline, and group 3: infected mice with *C. albicans* and treated with EG-AgNPs. Mice were treated daily for 5 days starting after day 5 of infection by pipetting 50ul of Ag (50 ug/mL) on the dorsum of the tongue of anesthetized mice. The control-infected group received sterile phosphate-buffered saline (PBS) using the same oral treatment method. Evaluation of OC treatment was performed after 4 days of treatment. Colony forming units (CFU/mL) of *C. albicans* were recovered from the oral cavity by swabbing the tongue with a cotton swab then putting it in sterile saline to be vortexed thoroughly. A serial dilutions of the cell suspension were then plated on Sabouraud dextrose agar plates and cultured at 37 °C for 24 h, followed by counting of the colonies.

### 4.12. Histological Analysis of Tongue

Quantification of oral infection of the tongue’s fur and squamous layer was performed according to method described by [85]. Tongues were taken from sacrificed animals fixed in 20% formalin solution and embedded in paraffin blocks. Micro sections were cut from the paraffin block and stained first with hematoxylin and eosin stain (HE) for histological examination and then stained periodic acid Schiff (PAS) stain for yeast detection.

### 4.13. Statistical Analysis

Values were expressed as mean ± SD (standard deviation) of at least three independent experiments. Power calculation was performed for two samples using unpaired Student’s *t*-test (two-tailed), assuming equal variation in the two groups. Differences were considered statistically significant at * *p* < 0.05, and ** *p* < 0.005.

## 5. Conclusions

The current study revealed that silver nanoparticles can be biosynthesized using a very simple, inexpensive, eco-friendly method that uses *E.* *glaucophyllum* leaf extract (EG-AgNPs). The TEM analysis showed that the sizes of the synthesized AgNPs ranged from 50 to 100 nm. This technique revealed that the leaf extract can be used as an efficient stabilizing reducing and capping agent for the synthesis of AgNPs, thereby providing stability to the silver nanoparticles. The biosynthesized AgNPs were crystalline in nature as evident from the XRD spectral analysis. Our results revealed the significant inhibitory effect of EG-AgNPs on the growth, dimorphic transition, biofilm formation, and the production of hydrolytic enzymes of *C. albicans*. EG-AgNPs demonstrated no sign of cytotoxicity on an in vitro mammalian cell line. Interestingly, topical application of EG-AgNPs on the infected tongue of the oral candidiasis (OC) mouse model showed a significant reduction in the candidal tissue invasion, in association with marked low scare and hyphal counts of *C. albicans* as compared to non-treated control mice group. Thus, our data proved EG-AgNPs as promising antifungal drugs for the treatment and/ or prevention of OC via controlling the pathogenesis of *C. albicans* by inhibiting the key virulence factors.

## Figures and Tables

**Table 1:** Anti-candidal activity of EG-AgNPs.

Concentration (µg/mL)	Antifungal Agent		
	DMSO	AMB	EG-AgNPs
	IZD (mm)		
0	_a_0^a^ ± 0.0	_a_0^a^ ± 0.0	_a_0^a^ ± 0.0
6.25	_a_0^a^ ± 0.0	_b_4^c^ ± 0.6	_b_10^b^ ± 0.7
12.5	_a_0^a^ ± 0.0	_c_9^c^ ± 0.3	_c_16^b^ ± 0.4
25	_a_0^a^ ± 0.0	_d_15^c^ ± 0.4	_d_21^b^ ± 0.9
50	_a_0^a^ ± 0.0	_e_17^a^ ± 0.8	_a_0^b^ ± 0.6
100	_a_0^a^ ± 0.0	_a_0^a^ ± 0.0	No growth
200	_a_0^a^ ± 0.0	No growth	
400	_a_0^a^ ± 0.0		

IZD = Inhibition zone diameter (mm). Data are expressed as the mean zone of inhibition in mm followed by SD. Superscript and Subscript letters showed the significant different according to ANOVA and Duncan’s multiple range tests.

**Table 2:** Effect of EG-AgNPs against dimorphic transition of *C. albicans*.

Antifungal Agent	YF Count (cell/mL)	FF Count (cell/mL)	% of Dimorphism
**DMSO**	98^a^ ± 4.0	1910^c^ ± 4.0	94.86 ± 1.7
**AMB**	155^b^ ± 5.0	275^a^ ± 1.5	43.63 ± 0.9
**EG-AgNPs**	312^c^ ± 6.0	715^b^ ± 2.0	56.36 ± 1.4

The values with different superscript letters are significantly different as analyzed by ANOVA and Duncan’s multiple range tests. YF: Yeast form FF: Filamentous form % of dimorphism= FF-YF/FF × 100.

## Data Availability

The data presented in this study are available on request from the corresponding author.

## Supplementary Materials

The following supporting information can be downloaded at: https://www.mdpi.com/article/10.3390/molecules27134221/s1, Figure S1: Histogram and Gaussian fit of EG–AgNPs size distribution by intensity from dynamic light scattering measurement.

## Sample Availability

Samples of the compounds are available upon request from the authors.
