# Supplementary material for: Therapeutic Effect of Green Synthesized Silver Nanoparticles Using Erodium glaucophyllum Extract against Oral Candidiasis: In Vitro and In Vivo Study
Source: Molecules. 2022 Jun 30;27(13):4221. doi: 10.3390/molecules27134221 (PMC9267989; doi:10.3390/molecules27134221)
Supplement: Supplementary file 1 [file molecules-27-04221-s001.zip › molecules-1767878-supplementary.pdf]

Supplementary Figure S1

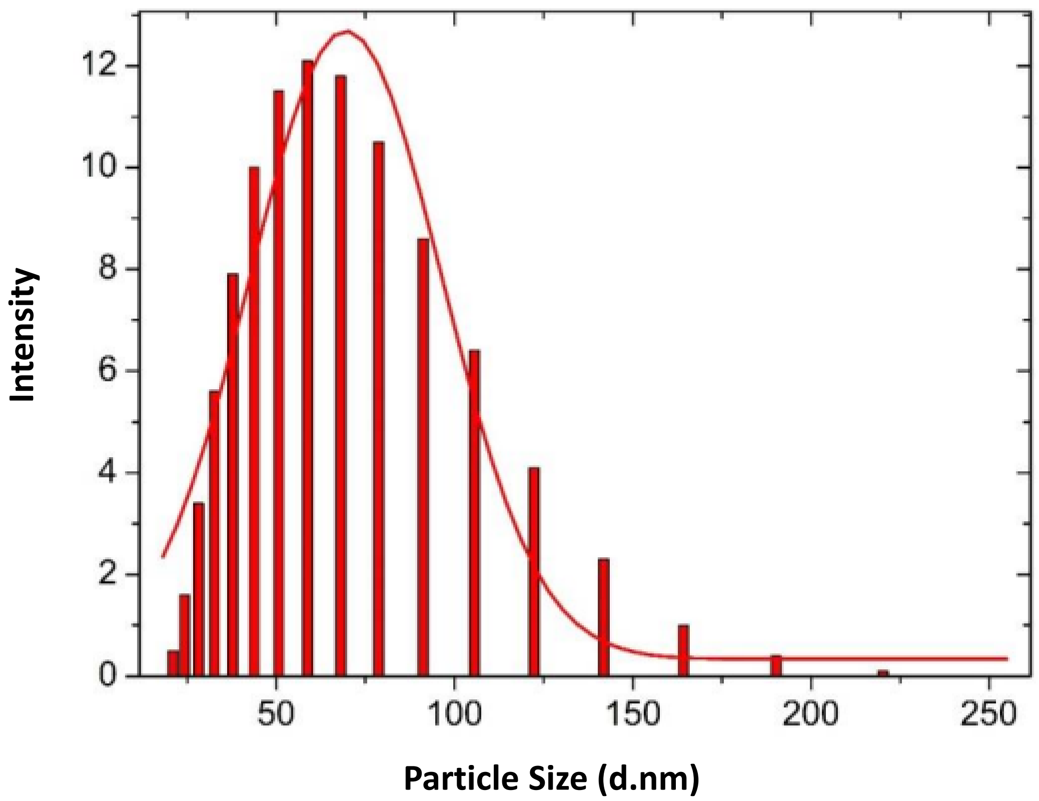

Supplementary Figure S1. Histogram and Gaussian fit of EGAgNPs size distribution by intensity from dynamic light scattering measurement.
